# High sensitivity and wide response range artificial synapse based on polyimide with embedded graphene quantum dots

**DOI:** 10.1038/s41598-023-35183-8

**Published:** 2023-05-20

**Authors:** Lijie Kou, Nan Ye, Anjam Waheed, Rahmat Zaki Auliya, Chaoxing Wu, Poh Choon Ooi, Fushan Li

**Affiliations:** 1 School of Computing and Information Sciences, Fuzhou Institute of Technology, Fuzhou, 350506 People’s Republic of China; 2 Institute of Microengineering and Nanoelectronics (IMEN), Universiti Kebangsaan Malaysia, 43600 Bangi, Selangor Malaysia; 3 School of Physics and Information Engineering, Fuzhou University, Fuzhou, 350002 People’s Republic of China

**Keywords:** Materials science, Physics

## Abstract

Artificial electronic synapses are commonly used to simulate biological synapses to realize various learning functions, regarded as one of the key technologies in the next generation of neurological computation. This work used a simple spin coating technique to fabricate polyimide (PI):graphene quantum dots(GQDs) memristor structure. As a result, the devices exhibit remarkably stable exponentially decaying postsynaptic suppression current over time, as interpreted in the spike-timing-dependent plasticity phenomenon. Furthermore, with the increase of the applied electrical signal over time, the conductance of the electrical synapse gradually changes, and the electronic synapse also shows plasticity dependence on the amplitude and frequency of the pulse applied. In particular, the devices with the structure of Ag/PI:GQDs/ITO prepared in this study can produce a stable response to the stimulation of electrical signals between millivolt to volt, showing not only high sensitivity but also a wide range of “feelings”, which makes the electronic synapses take a step forwards to emulate biological synapses. Meanwhile, the electronic conduction mechanisms of the device are also studied and expounded in detail. The findings in this work lay a foundation for developing brain-like neuromorphic modeling in artificial intelligence.

## Introduction

A memristor is considered the fourth basic circuit element after a resistor, inductor, and capacitor. In 1971, Chua^1^ first proposed the concept of memristor based on the principle of symmetry, then Strukov^2^et al. confirmed it through experiments in 2008. The memristor research provides a feasible route for developing a new computer architecture that integrates information storage and processing, which breaks through the bottleneck of traditional von Neumann architecture. Memristors have several advantages, such as non-volatility, high speed, low power consumption, simple structure, and easy integration^3,4^. As such, it has shown broad application prospects in a new generation of high-density non-volatile memory, neuromorphic artificial intelligence, high-speed logic operations, and secure communications^5–7^.To date, the functions of biological synapses that are simulated by memristors comprise spike-timing-dependent plasticity (STDP), long-term potentiation/depression (LTP/LTD), short-term potentiation/depression (STP/STD), and paired-pulse facilitation (PPF). Memristors that can simulate these functions are often called electronic synapses (e-synapses). E-synapses are expected to mimic biological synapses comprehensively, which is crucial to realizing brain-like neuromorphic information processing and artificial intelligence (AI) computing at the component level^8,9^.

Even though some promising achievements have been reported, nevertheless, the current memristors study still encountered some problems, typically device instability, discrete parameter distribution, and insufficient mechanical strength and durability for wearable flexible devices, resulting in a gap from commercial use standards^10,11^. Usually, the preparation process adopted to improve the device performance increases the process complexity and is unsuitable for high-volume manufacturing. It is also worth noting that the high sensitivity and wide receptive field of biological synapses to external microstimuli enable biological synapses to speed up information processing more than computers in terms of individual safety, information processing, and reduction of power consumption. However, e-synapses reported in previous literature can only respond to a limited range of electrical signals, especially those that can react to microstimulation are rarely reported^12^. Therefore, for neural computing and AI applications, it has become preferable to use materials with high mechanical properties and chemical stability to obtain flexible memristors with stable performance, high sensitivity, and a comprehensive response range through simple and easy-to-operate processes^13,14^.

Consequently, this work deliberately designed a simple three-layer structure device using polymer and quantum dots composite as the functional resistive material. Considering the advantages of low cost, facile fabrication process, industrial mass production suitability, solution process technique, specifically spin coating, has been used to deposit the memristor resistive layer. Compared with inorganic oxide materials, polymer materials have attracted much attention due to their advantages that other inorganic materials cannot surpass, especially low density, wear resistance, corrosion resistance, and high flexibility^15,16^. Among them, polyimide (PI) has strong mechanical and chemical stability, high electrical reliability, exhibits unique electrical properties, and high flexibility in a variety of wearable devices^17,18^. In addition, PI that combines the above advantages is easy to synthesize and hence has broad potential in research and practical applications, either as functional materials or as supporting substrates^19^. Thus, in this study, PI was used as the host functional material to lay the foundation for the device’s high flexibility and chemical stability.

Graphene quantum dots (GQDs) also possess high thermal, chemical, and electrical stability characteristics. Hence, they are suitable to be incorporated into the polymer host to form uniformly distributed charge traps in the device, which can effectively improve the discrete distribution of memristor parameters^20^. In addition, ascribed to the ultra-abundant and sensitive surface electron trapping ability resultant from the high specific surface area of GQDs, the e-synapse applying PI:GQDs composites is expected to improve the response-ability to micro-stimulation signals. Furthermore, dispersing GQDs into a polymer matrix might be another advantage to improve the device stability as compared to the conductive polymer poly(3,4-ethylenedioxythiophene) polystyrene sulfonate^21^. This is due to the high insulation property of PI material. The solution process technique is a timely, efficient method compared to the atomic layer deposition (ALD) of the insulator layer and yet yielded the equivalent stability^22^. A solution process is also a wise option for high-throughput fabrication as in actual application. The memristor device dimension is about micron scale to develop the excellent potential for high integration density^23,24^.

## Materials and methods

### Nanocomposite preparation

P-phenylene biphenyltetra carboxamide (BPDA-PDA) was dissolved in dimethyl formamide (DMF) with a concentration of 1 wt% to prepare a PI precursor solution. Then, the solution was stirred under magnetic agitation for 2 h and left for 24 h. 1 mg/ml GQDs in DMF solution purchased from Nanjing XFNANO Materials TECH Co. (CAS: 7440-44-0). The prepared PI precursor and GQD suspension were mixed at different volume ratios of 10:1, 20:1, and 50:1 for further optimization. Finally, the prepared mixed solutions were sonicated for 10 min to ensure homogenous distribution of GQDs in the PI matrix.

## Fabrication of memristor devices

Firstly, a 3 cm × 3 cm glass coated with indium-tin-oxide (ITO) was sequentially ultrasonically cleaned with deionized water, acetone, and isopropanol for 20 min, respectively. After cleaning, it was dried in a 60 °C vacuum oven, followed by 15 min of oxygen plasma treatment. Next, the prepared PI:GQDs mixture was spin-coated at 300 rpm for 5 s, followed by 3000 rpm for 40 s on the cleaned ITO/glass to form a ~ 30 nm nanocomposite layer. Subsequently, the deposited film was heat treated at 200℃ for 1 h. Finally, the Ag top contacts were formed on the nanocomposite layer using a 1 mm diameter shadow mask under the 3 × 10^–3^ Pa vacuum thermal evaporation. In the same way, the device of PI without GQDs doping was prepared as a reference sample.

## Characterizations and measurements

GQDs were characterized by transmission electron microscopy (TEM; FEI Titan ETEMG2) and atomic force microscope (AFM; Bruker multimode 8). The cross-sectional image of the device was obtained by the ZEISS MERLIN field emission scanning electron microscope (FESEM). The device’s electrical performance was tested by the Keithley 4200 semiconductor characterization system. All tests were conducted at room temperature and ambient conditions.

## Results and discussion

### Device structure and GQDs characterization

**Figure 1:**
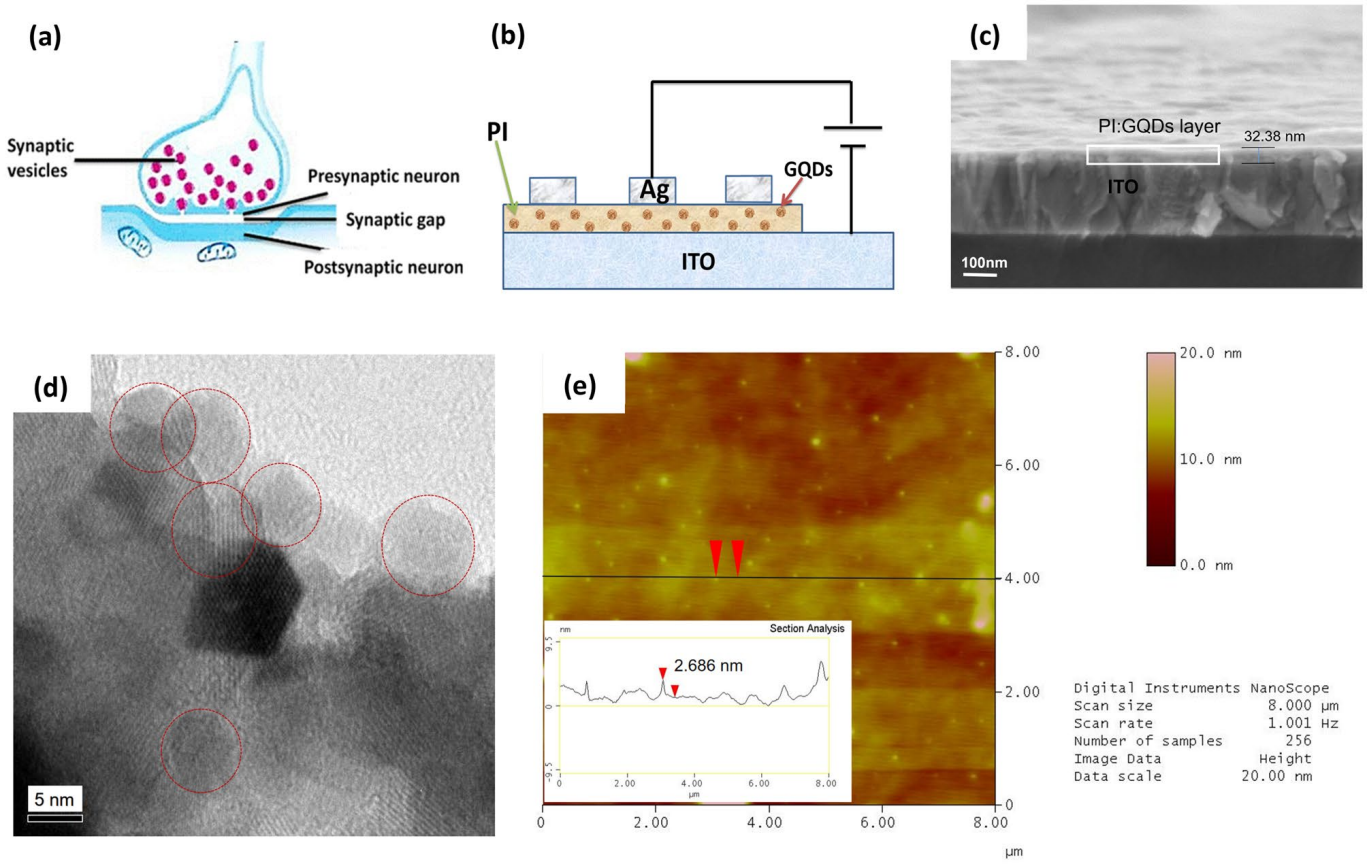
Schematic diagram of (**a**) biological synapse, and (**b**) fabricated memristor device in the structure of Ag/PI:GQDs/ITO. (**c**) FESEM cross-sectional structure of a fabricated device. (**d**) TEM, and (**e**) AFM images of GQDs.

A biological synapse is the contact site of two neurons, composed of a presynaptic membrane, synaptic gap, and postsynaptic membrane, as shown in Fig. 1a. When a synapse is stimulated by the activity of the anterior or posterior neuron, its synaptic weight changes, and the information is transmitted from one neuron to another through the aforementioned functional connections. In parallel, it updates the synaptic weight, which facilitates transportation^25,26^ Fig. 1b shows the Ag/PI:GQDs/ITO memristor structure of this work. Figure 1c shows the device's 30 nm memristive layer FESEM cross-sectional image. The memristive layer is the key to the synaptic storage and processing of information. The resistivity of the memristor layer forms by dispersing the GQDs uniformly in the PI matrix. When the charges are injected from the electrode, the memristor's conductance changes and demonstrates different response phenomena similar to biological synapses known as an e-synapse. Figure 1d shows the TEM image of the GQDs. It is found that the size distribution of GQDs adopted in this work is around 6–9 nm, as marked with red circles. Meanwhile, Fig. 1e shows AFM observation of the GQDs. The inset in Fig. 1e shows that the thickness of GQDs is about 2–3 nm. The size of the quantum dots determines the control of the charge transport in the thin film, resulting in a time-dependent memristive effect.

### Electrical characterizations

**Figure 2:**
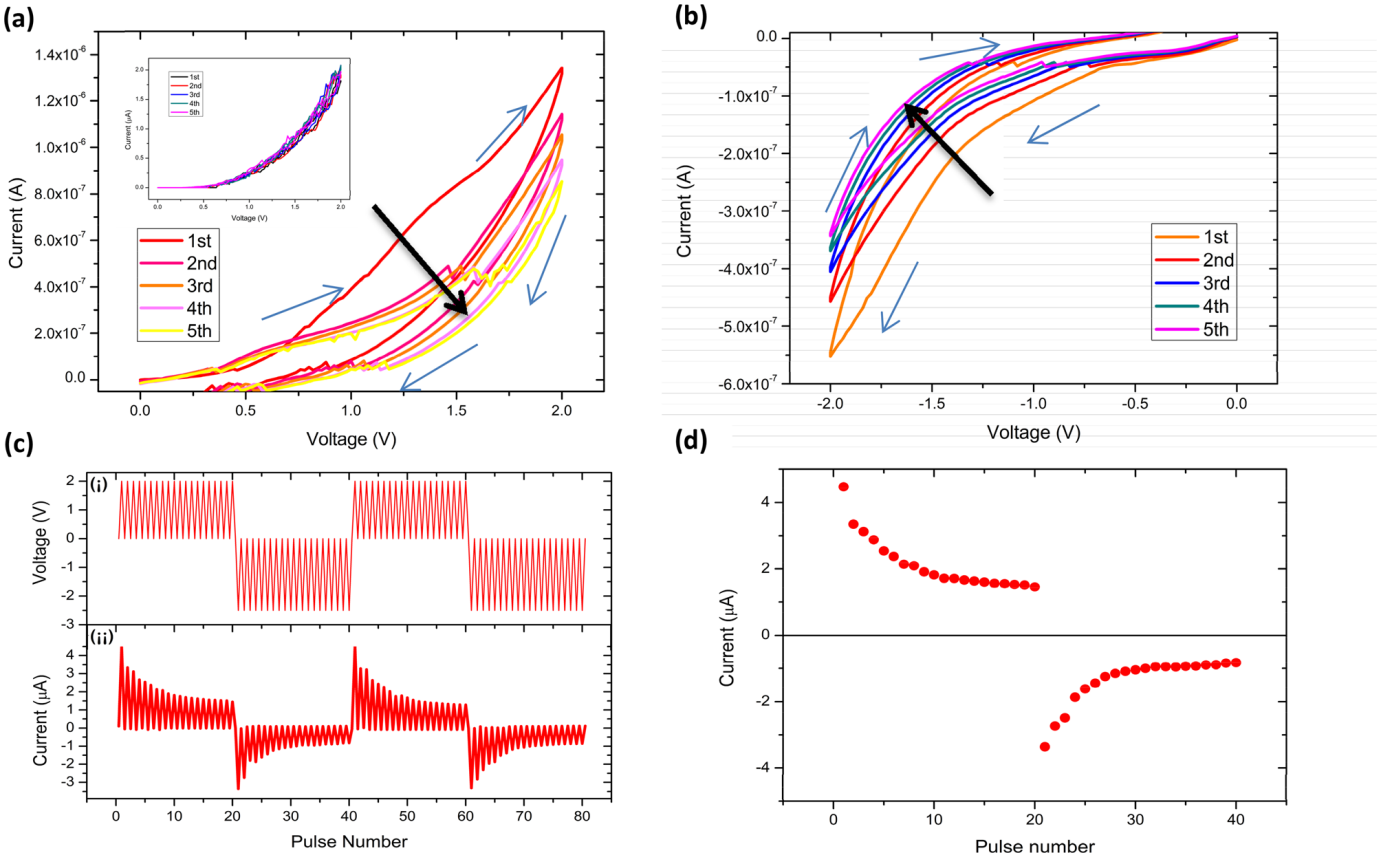
Various electrical characteristics of the fabricated devices. I-V curves of the device under repeated application of (**a**) positive, and (**b**) negative triangle-wave voltage sweeps. The inset of (a) shows the ignorable I–V for the reference sample. (**c**) (i) Applied pulse voltage waveform, and (ii) the device’s response under the pulse voltage simulation. (**d**) Variation of current value in one cycle of the impulse response.

The incorporation of quantum dots significantly affects the memristive performance of fabricated memristors. If the concentration of GQDs incorporated is too low, it is difficult to achieve effective traps. Conversely, high GQDs concentration may change the coordination ratio of the PI precursor solution, resulting in the deterioration of the PI precursor solution and the difficulty of forming a stable PI film. It was found that the device outperformed when the volume ratio of PI precursor to GQDs was 20:1. Figure 2a shows the device’s performance under repeated application of 0 to 2.0 V triangle-wave voltage sweeps to observe the presynaptic. Under the action of the first sweep, the device shows a high conductance state. Then each scan will make the conductance value smaller than the previous; the device conductance decreases gradually with the increased scanning repetition and does not observe the apparent abrupt change of current. After multiple scans, the resistance value of the device gradually switches to a low conductivity state. Figure 2b shows that when the reverse negative voltage is applied for the first scan, the conductance value of the device returns to the reverse high conductance state again, indicating that the device’s high and low conductance states can be set and reset repeatedly. In the following several times of reverse voltage scanning, the conductance state of the device gradually decreases, implying phenomena similar to positive voltage scanning. Under the continuous stimulation of direct current voltage, the conductance state of the fabricated memristor gradually changes, as shown with the arrow in Fig. 2a and b. That means the synaptic weight of the e-synapse gradually changes, similar to the performance of biological synapses. The reference sample without GQDs exhibited a pure resistance effect and did not exhibit a memristive effect, as shown in the inset of Fig. 2a. Figure 2b shows that when the reverse negative voltage is applied for the first scan, the conductance value of the device returns to the reverse high conductance state again, indicating that the device’s high and low conductance states can be set and reset repeatedly. In the following several times of reverse voltage scanning, the conductance state of the device gradually decreases, implying phenomena similar to positive voltage scanning. Under the continuous stimulation of direct current voltage, the conductance state of the fabricated memristor gradually changes, as shown with the arrow in Fig. 2a and b. That means the synaptic weight of the e-synapse gradually changes, similar to the performance of biological synapses. The reference sample without GQDs exhibited a pure resistance effect and did not exhibit a memristive effect, as shown in the inset of Fig. 2a. The 200 °C heat treatment after a spin-coating process of PI:GQDs mixture is crucial to ensure good electrical insulation properties. Therefore, it rarely appears short circuits between ITO and Ag electrodes during electrical tests.

Figure 2c shows the current response of the device when 20 positive and negative pulses were alternately applied to the device. Due to the different contact resistances caused by the different top and bottom electrode materials, the circulation pulse test uses asymmetrical positive and negative voltage values. Consequently, the positive pulse voltage is set to 2 V, but the negative pulse voltage is set to 2.5 V, with a width of 0.1 s and interval of 1 s, as shown in Fig. 2c(i). In order to make a clear observation trend, Fig. 2d shows the change of current value under impulses in the first 40 pulses. It can be seen that the value of the current flowing through the device is gradually reduced under the action of the pulse voltage, indicating that the conductance state changes from high to low. When the reverse voltage pulse is applied, the device returns to the high current state. With the pulse numbers increasing, the device’s conductivity gradually decreases. The device can repeat the same electrical behavior by applying continuous positive and negative pulses.

### Synaptic STD, PPF, and LTP

**Figure 3:**
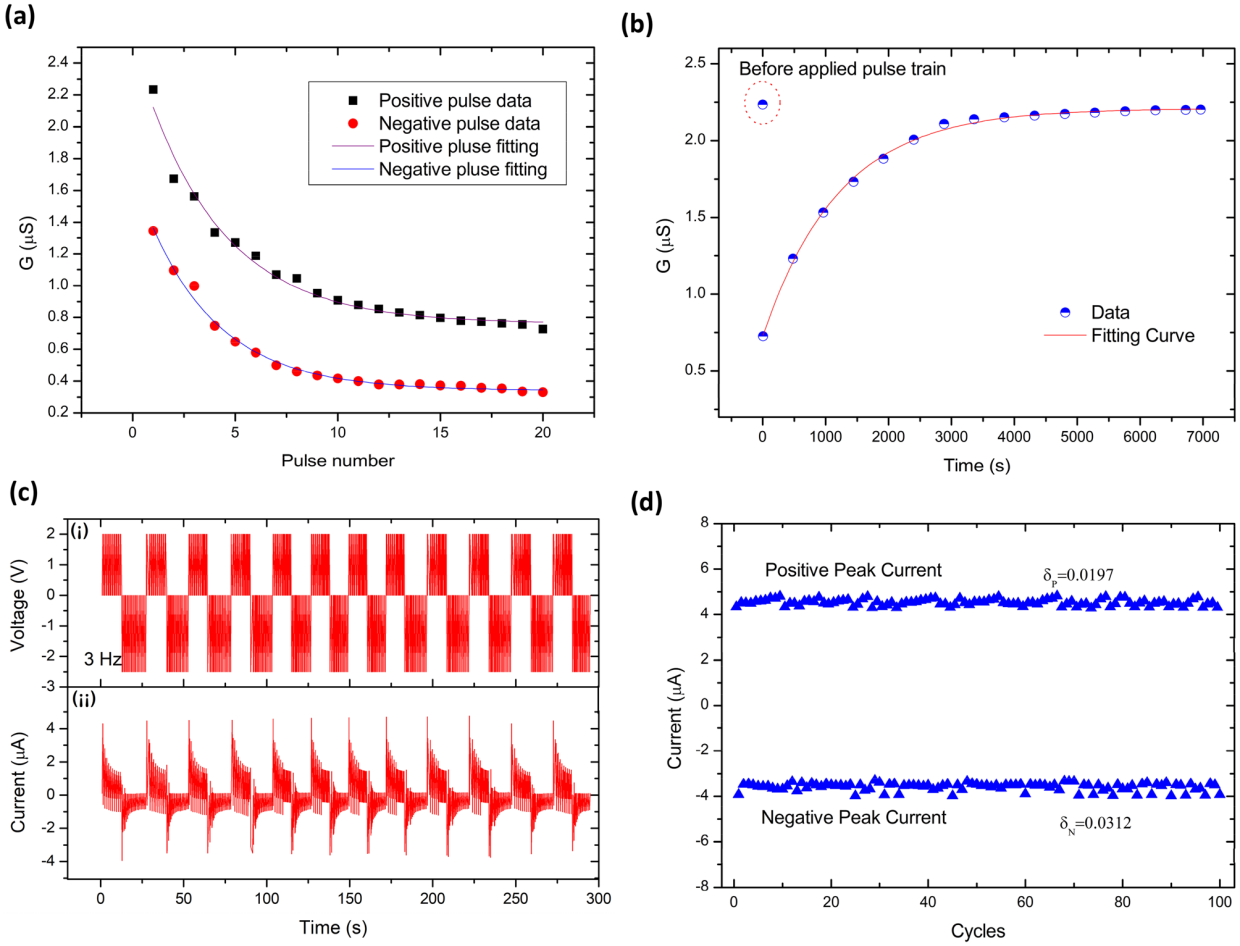
Synaptic plasticity of the fabricated memristor. (**a**) Data and curve fitting plots of positive and negative calculated conductance. (**b**) Forgetting curve of e-synapse under a single pulse of 0.1 V once every 8 min. (**c**) (i) Applied cyclic pulse, and (ii) device responses under 300 s continuously applied pulse. (**d**) The peak value of positive and negative current variance for 100 cycles.

The characteristic that synaptic weights change depending on the stimulus is called synaptic plasticity, which is the neurobiological basis of memory and learning in our brain, as well as the primary function of artificial synapses. Figure 3 shows the synaptic plasticity of the memristor based on Ag/PI:GQDs/ITO structure. Figure 3a plots the calculated conductance based on the peak value of voltage and current of each pulse and the curve fitting for positive and negative pulses. It can be observed that the subsequent conductance of the fabricated device under each positive pulse stimulation is lower than the previous value, which means the synaptic weight changes. The likewise trend is also observed for the negative pulses. Therefore, the fabricated device can remember the previous learning state when it is being stimulated again, indicating that the Ag/PI:GQDs/ITO structure exhibits short-term inhibition learning that resembles STD. In addition, it can be seen that when continuous positive or negative pulses are applied to the fabricated memristor, it shows the same trend of conductance change. The conductance value drops rapidly during the first ten pulses, then gradually slows down and eventually reaches saturation. Such a change process indicates the exponential learning process of the memristor, which is similar to the biological learning process. The difference between positive and negative conductance values is mainly due to the different work functions of the Ag and ITO electrodes. Hence, the electrons need different excitation energy to overcome the same PI barrier when voltage is applied to the device in different polarities. As a result, the memristor shows an asymmetrical conductance.

The bio-synaptic memory behavior is divided into short-term memory and long-term memory. We also conducted the forgetting memory characteristics test of the memristor fabricated, as shown in Fig. 3b. Initially, the device was recording in the high-conductance state at 2.25 µS. After the device had completed the learning by positive pulse stimulation with pulse voltage at 2.0 V, a width of 0.1 s, and an interval of 1 s, it switched to the low-conductance state of 0.75 µS. Afterward, no stimulation was applied. The device is read once every 8 min with a pulse of 0.1 V to investigate the conductance state. Since the read-out pulse was so short and low in magnitude, the effect of learning by the read-out pulse is neglectable. Notably, the conductance value rises slowly, and the device almost returns to the initial state after about 1 h. This behavior resembles the forgetting performance of the human brain, referring to the forgetting memory curve proposed by Ebbinghaus^27^, which describes the decline in the human brain's probability of recalling memories over time. The exponential function, R = exp(− t/S), can be used to fit the conductance data of the memristor forgetting process, where R, t, and S symbolize memory retention, time, and the relative strength of the memory, respectively^28^. Figure 3b shows the well-fitted curves of data points and forgetting trends. Figure 3c shows the 300 s current behavior as a consequence of the continuously applied pulse from 2.0 to − 2.5 V. The device exhibited no significant degradation up to 100 cycles, as shown in Fig. 3d. The peak value of positive and negative current variance for all the cycles was estimated to beδ_P_ ~ 0.0197 and δ_N_ ~ 0.0312, inferring that the absolute change is visibly stable.

**Figure 4:**
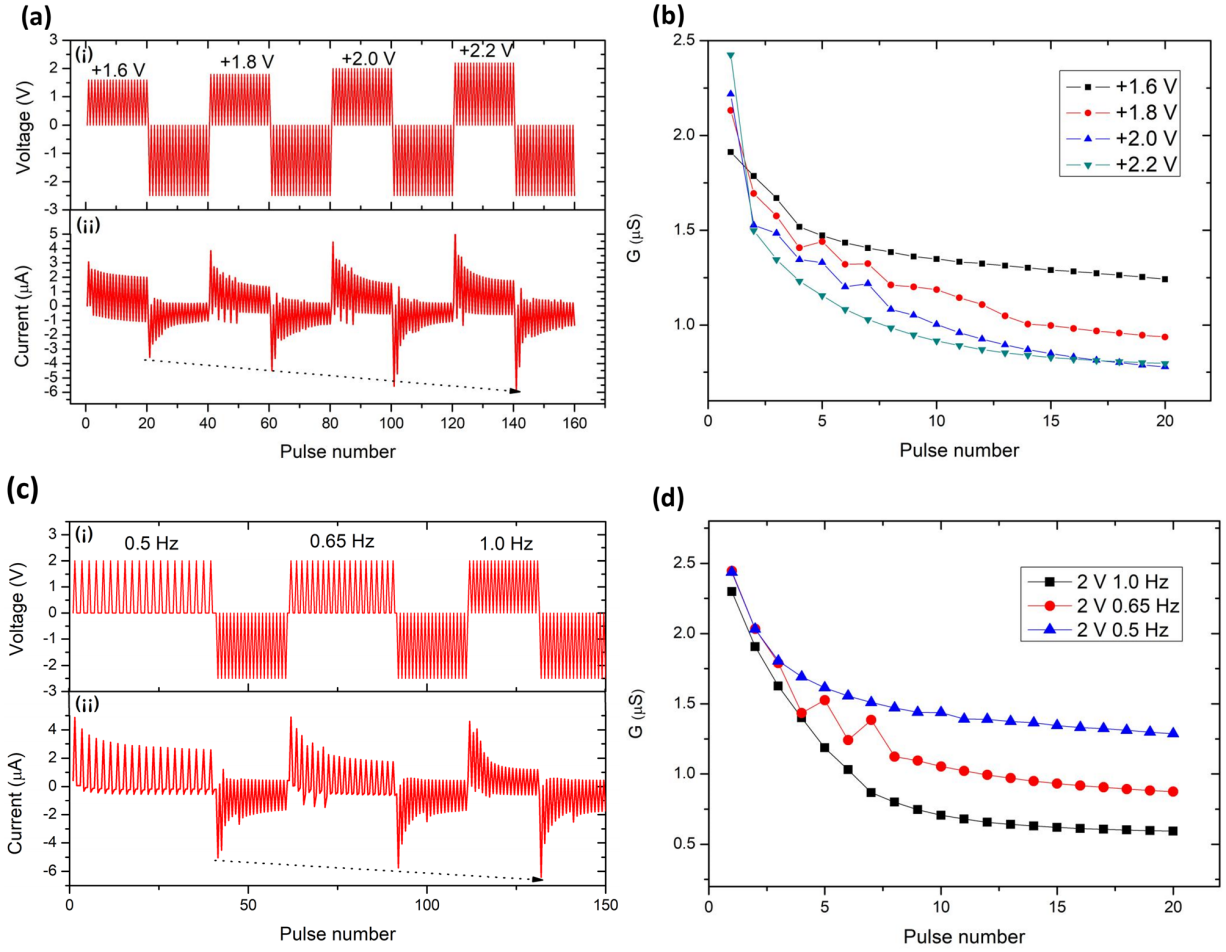
Amplitude and frequency dependence of prepared memristor device. Different (**a**) (i) amplitudes, and (**c**) (i) frequencies of the applied pulse. The current response of the devices under the stimulation of different (**a**) (ii) amplitudes, and (**c**) (ii) frequencies. Equivalent conductance value change curves under pulse stimulations of different (**b**) amplitudes, and (**d**) frequencies.

In some biological neural circuits, the plasticity of synapses shows not only time-series dependence but also amplitude and frequency dependence. Hence, the memristor prepared in this work was also tested for amplitude and frequency dependence, as shown in Fig. 4. Figure 4a(i) shows the applied waveform diagram. To test for amplitude dependence, + 1.6 V was applied to the device for the first 20 pulses, followed by 20 negative pulses at − 2.5 V. The applied negative pulse can read the resistance state of the device and reset the device simultaneously. For the next period, the amplitude of 40–60 positive pulses was increased to + 1.8 V and set with the same negative applied pulse from 60 to 80. In the following third and fourth periods, the positive pulse train maintained an increment step of 0.2 V relative to the previous period. In contrast, the reverse pulse train always used the same amplitude. Resultantly, in Fig. 4a(ii), the obtained negative current measurement is directly proportional to the amplitude of the positive pulse, indicating the synapse’s level of strong learning depth. This suggests that synaptic learning depth depends on the intensity of presynaptic stimuli. Increasing stimulation leads to continuous and deep learning. Figure 4b shows the derived conductance plots with the change of presynaptic pulse amplitude, as shown in Fig. 4a(ii). The device demonstrates STD behavior under different amplitude pulse stimulation. Note that the final conductance of the synapse is inversely proportional to the strength of the applied pulse amplitude. In another way, the saturation point reflects the level of learning depth.

Figure 4c(i) shows the frequency dependence of synaptic learning at the constant 2.0 V and − 2.5 V of the 20-pulse train. Positive pulse trains of different frequencies in three periods were tested at 1.0 Hz, 0.65 Hz, and 0.5 Hz, respectively. At the same time, the subsequent three periods of negative pulse train were set at 1.0 Hz to read and reset the device’s current state. Generally, in Fig. 4c(ii), at the constant 2.0 V pulse train, the measured current at low frequency decreases gradually, resulting in a higher current relative to the high-frequency cycle current that decreases rapidly. As such, the rate of the current decreases correlates proportionally with the frequency of the applied pulse trains. The frequency dependence behavior implies that the interval between two positive pulses changes, thus resulting in a different degree of change in the postsynaptic response, that is, the PPF of synapses. PPF occurs when two equal pulses are separated in time, and the postsynaptic response (current) is measured. The current response on the second pulse should depend on the time distance between the two pulses. This mimics the behavior of biological synapses, where pulses right after one another facilitate information transport.

In contrast, the current shows an apparent analogous decreasing trend for the three negative pulse trains that fixed at 1.0 Hz with the constant − 2.5 V. However, the initial absolute maximum current reading for this increase was recorded to be 5.033 µA, 5.736 µA, and 6.407 µA, after 0.5 Hz, 0.65 Hz, and 1.0 Hz, respectively. It is worth noting that the starting point of the second negative period (5.736 µA) is higher than the first negative period (5.033 µA). Likewise, the onset current of the third negative period (6.407 µA) is higher than the second period (5.736 µA), as indicated by the arrow in Fig. 4c(ii). This behavior can be described as LTP, a significant reflection of synaptic plasticity, which is an activity-driven long-lasting increase in the efficiency of excitatory synaptic transmission following the delivery of a brief, high-frequency train of electrical stimulation. The reason could be that the frequency of the applied positive pulse train increases over the three cycles, implying that a higher positive pulse frequency results in a faster reduction in the forward current of the device. In other words, a faster learning rate, thus resulting in a higher level of negative learning depth achieved. This behavior is consistent with the biological synaptic learning process. Figure 4d shows the fabricated devices’ conductance curves derived from Fig. 4c under the stimulation of three different frequencies at a 2.0 V pulse train. It is clear that at 1.0 Hz, the memristor rapidly changes from a high conductance state to a low conductance state. When the frequency of the applied pulses decreases sequentially to 0.65 Hz and 0.5 Hz, the changing trend of conductance was slighter compared to the response under 1.0 Hz frequency stimulation. Subsequently, the conductance behavior of the devices switches to a slow-changing saturation state after the initial rapid change. The three saturation states of 1.0 Hz, 0.65 Hz, and 0.5 Hz readings are attained at 0.594 µS, 0.874 µS, and 1.287 µS, respectively. Markedly, the saturation conductance reading under higher frequency stimulation is lower, equivalent to a more considerable synaptic weight for e-synapses. On the contrary, the synapse saturation state under low-frequency stimulation is smaller.

Paired-pulse facilitation (PPF) is a form of synaptic plasticity in which synapses can change their ability to transmit information, which is vital in the nervous system. PPF is to help regulate communication between neurons and influences important cognitive processes, especially learning and memory storage. In this test, the device was set to an initial high conductance state with consistent conductance values, followed by two consecutive 2 V pulses time difference (∆t) of 0.35, 1.0, 1.5, 2.0, and 2.5 s, respectively. The conductance difference can be calculated by recording the initial and final conductance, remarked as G_i_ and G_f_, respectively. Therefore, the relative rate of change in conductance is estimated by {∆G = [|(G_f_ − G_i_)|/G_i_] × 100%}, which is the PPF index, as shown in Fig. 4e. As the time interval between paired-pulse decreases, the rate of change of the device conductance increases, resembling the learning intensity of a synaptic neuron increases with the shorter time interval between successive stimuli. Conversely, as the paired-pulse interval increases, the rate of conductance change of the device decreases rapidly, which is consistent with the fact that the learning enhancement of biological synaptic neurons becomes less effective for the longer interval between learning stimuli. This is also a manifestation of short-term potentiation.

### High sensitivity and wide response range

**Figure 5:**
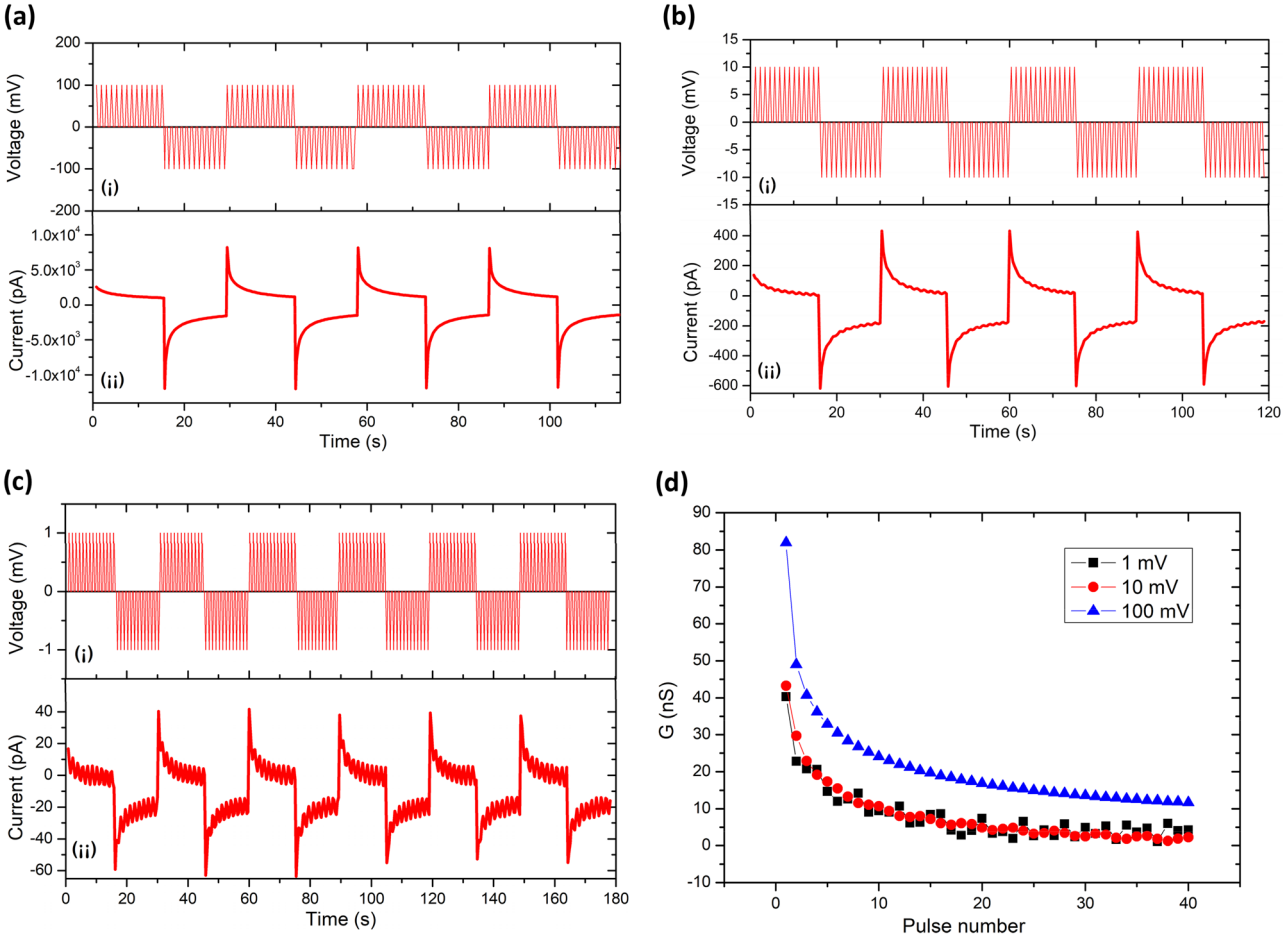
High sensitivity and wide sensing range performance. Applied pulse voltage of (**a**) (i) 100 mV, (**b**) (i) 10 mV, and (**c**) (i) 1 mV. The current response of the fabricated devices under the stimulation of (**a**) (ii) 100 mV, (**b**) (ii) 10 mV, and (**c**) (ii) 1 mV (**d**) Conductance values comparison under the different magnitudes of pulse voltage stimulation.

The synapses in the biological nervous system usually have high sensitivity and a wide response range, enabling the biological system to respond nimbly and intelligently to slight environmental changes. Figure 5 shows the sensitivity test of the fabricated memristor based on Ag/PI:GQDs/ITO structure. Notice that in the obtained results in Section "Synaptic STD, PPF, and LTP", the voltage amplitude of the pulse signal applied to the device was on the order of volts, which is not in the range of microstimulation. Therefore, the amplitude of the applied pulses was set to ± 100 mV, ± 10 mV, and ± 1 mV, respectively, to examine the device’s sensitivity and response range. Under the impulse stimulation of mV order of magnitude, the device still shows prominent memristor characteristics. In Fig. 5a(i), the amplitude of the applied pulse voltage sequence for each positive and negative cycle was set at + 100 mV and − 100 mV with a width of 0.1 s and interval of 1.0 s. Figure 5a(ii) shows that the fabricated device still exhibited a good and stable STD under the excitation of ± 100 mV pulse voltage. Then, to further investigate the sensitivity of the devices, the amplitude of the applied pulse voltage was further decreased to ± 10 mV and ± 1 mV, as shown in Fig. 5b(i) and c(i), respectively. Again, the memristor also exhibited promising stable STD behavior in Fig. 5b(ii) and c(ii). More importantly, the state transition trend is consistent with the performance under higher voltage stimuli. Additionally, the device can maintain significant memristive behavior even though the pulse amplitude of ± 1 mV has been reduced by 10^–3^ compared with the 2.0 V applied pulse in Fig. 2c(i). The obtained current in Fig. 5c(ii) shows a low level of conductance jitter from noise signals, which could be ignored as it did not affect the current trend of the device. Alternatively, the noise can be filtered for actual precise application. Figure 5d shows the comparison conductance plots derived from Fig. 5a–c. The device maintains a consistent fundamental change in synaptic weight under the stimulation of different pulse signals, from 1 mV to 2.0 V, with the voltage amplitude variation span in 10^3^ orders of magnitude. This behavior inferred the fabricated memristor based on Ag/PI:GQDs/ITO structure has ultra-high sensitivity to mV-level voltage and a wide response range.

### Working mechanism

**Figure 6:**
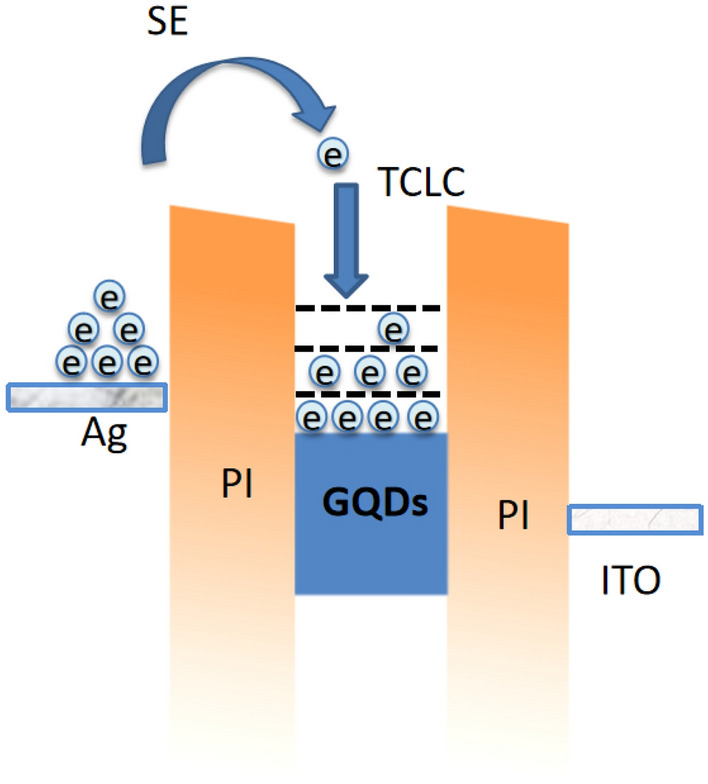
Possible charge transportation occurred through the fabricated memristor device under the applied voltage.

According to the literature, the proposed working mechanisms of memristor mainly include conductive wire^27,28^, electron capture^29,30^, and valence state transition^31^. For this study, considering that GQDs have various energy levels and unique surface areas, the charge trapping and storage capabilities of GQDs were designed and expected to impact charge transport significantly. Figure 6 illustrates the possible charge transport process of the prepared memristor device under applied voltage. First, the I-V curve fitting method was performed to analyze the charge transport process and memristive mechanism of the fabricated device. Then, the possible transport mechanisms were speculated based on the thickness of the fabricated devices. In Fig. 6, note that the occurrence of a thermionic emission current at low voltages is a typically observed mechanism and is commonly related to thermally-generated electrons. Subsequently, under the action of a forward bias voltage, Schottky emission is likely to take place as the I–V data in Fig. 2a fitted well ln(I/T^2^) α V^1/2^, where T is the temperature. In this event, the electrons are first emitted from the Ag electrode to overcome the PI dielectric barrier^32^. At this time, the macroscopically measured conductance value is the highest since electrons can be easily emitted and trapped in the various energy levels of GQDs^33,34^.

The transportation mechanism is switched to obey trapped-charge limited current (TCLC) when the electrons are rapidly captured in the trapping center formed by the uniformly dispersed GQDs. The obtained slope value is ~ 2.7, indicating the emergence of TCLC trapped^32^. The slope value is obtained through the double log plot of I–V data. As the trapped electrons gradually increase, a negative space charge region is formed throughout the device. A built-in electric field opposite the external electric field is established^22^, which hinders the continuous injection of external electrons from the electrode. Hence, the device begins transitioning from a high to low conductance state. Simultaneously, with the application of the forward pulses, electrons gradually fill up the multilevel energy traps of GQD, and the device exhibits a gradual decrease in the conductance state. The trapped electrons are rapidly released from the shallow traps when the device is reverse-biased, and the built-in electric field is immediately eliminated. Thus, the conductivity of the device returns to a high conductance state. Under the action of the subsequent reverse bias voltage, the device experiences a charge transport process similar to the forward bias process. The mechanism of the memristor forgetting process is that the multilevel energy bands of almost all GQDs are filled after the device enters a low-conductance state. Afterward, the bias is no longer applied, and the trapped charges will be released spontaneously. Eventually, the device will return to the initial high conductance state. The ultra-high sensitivity behavior can be correlated to the uniform distribution of GQDs in the polymer host, easy trapping, and release of charges of GQDs characteristics^17^. Since the amount of charge injected is related to the amplitude and frequency of the applied pulse, fabricated e-synapse exhibits plasticity dependent on both amplitude and frequency.

## Conclusion

Memristor based on a simple structure of Ag/PI:GQDs/ITO was prepared by an easy-to-operate solution process method in this work. The device exhibits time-dependent plasticity to the applied electrical signal. The synaptic weights of e-synapses change gradually with the applied electrical signals over time, and the e-synapses also show plasticity dependent on the amplitude and frequency of stimulations. The fabricated e-synapses demonstrate stable responses to stimuli from mV to V, exhibiting high sensitivity and a wide range. The conductive mechanism of the device is mainly derived from the charge capture mechanism of GQDs dispersed in PI. This PI:GQDs-based memristor exhibits excellent resistance-switching properties comparable to those fabricated from inorganic metal oxide materials. More importantly, it can further enhance various functions of simulated biological synapses, significantly improve the response range to electrical stimulation, and achieve an effective response to microstimulation. Hence, this study will pave the foundation for developing memristors in the neurological calculation.

## Data Availability

The datasets generated during and/or analysed during the current study are available from the corresponding author on reasonable request.
